# Characterization of Isoforms of the Lectin Isolated from the Red Algae *Bryothamnion seaforthii* and Its Pro-Healing Effect

**DOI:** 10.3390/md10091936

**Published:** 2012-09-04

**Authors:** Luiz Gonzaga do Nascimento-Neto, Romulo Farias Carneiro, Suzete Roberta da Silva, Bruno Rocha da Silva, Francisco Vassiliepe Sousa Arruda, Victor Alves Carneiro, Kyria Santiago do Nascimento, Silvana Saker-Sampaio, Valdemiro Amaro da Silva Jr., Ana Lúcia Figueiredo Porto, Benildo Sousa Cavada, Alexandre Holanda Sampaio, Edson Holanda Teixeira, Celso Shiniti Nagano

**Affiliations:** 1 LIBS, Integrated Laboratory of Biomolecules, Faculty of Medicine of Sobral, Federal University of Ceará, Fortaleza, CE 60020-181, Brazil; Email: ziullec@gmail.com (L.G.N.-N.); brunorocha747@gmail.com (B.R.S.); vassiliepe@gmail.com (F.V.S.A.); 2 BioMol, Laboratory of Biologically Active Molecules, Department of Biochemistry and Molecular Biology, Federal University of Ceará, Fortaleza, CE 60020-181, Brazil; Email: romulofc2603@gmail.com (R.F.C.), kyriasantiago@ufc.br (K.S.N.); bscavada@ufc.br (B.S.C.); 3 BioMar, Laboratory of Marine Biotechnology, Department of Fishing Engineering, Federal University of Ceará, Fortaleza, CE 60020-181, Brazil; Email: susiroberta@gmail.com (S.R.S.); sakersil@gmail.com (S.S.-S.); sampaioa@ufc.br (A.H.S.); 4 Department of Animal Morphology and Physiology, Federal Rural University of Pernambuco, Recife, Pernambuco 52071-030, Brazil; Email: valdemiroamaro@gmail.com (V.A.S.); analuporto@yahoo.com.br (A.L.F.P.)

**Keywords:** lectins, algal proteins, wound healing

## Abstract

Lectins are a structurally heterogeneous group of proteins that have specific binding sites for carbohydrates and glycoconjugates. Because of their biotechnological potential, lectins are widely used in biomedical research. The present study aimed to evaluate the healing potential of the lectin isolated from the marine red alga *Bryothamnion seaforthii* (BSL). The lectin was purified using ion exchange chromatography with DEAE cellulose and characterized using tandem mass spectrometry. For healing tests, skin wounds were induced in the dorsal thoracic region of mice. These animals were randomly divided into three groups and subjected to topical treatment for 12 days with BSL, bovine serum albumin and 150 mM NaCl. To evaluate the potential of each treatment, the animals were anesthetized and sacrificed on days 2, 7 and 12, respectively. The parameters evaluated included the wound area, the proportion of wound closure and the histological diagnosis. The wound closure was more effective with BSL (Postoperative Day 7 and 12) than controls. The luminal epithelium was completely restructured; the presence of collagen in the dermis and the strongly active presence of young skin annexes demonstrate the potential of treatment with BSL compared with controls. Our findings suggest that BSL has pro-healing properties and can be a potential medical process in the treatment of acute wounds.

## 1. Introduction

Cutaneous wounds are the result of a disruption of skin integrity and represent a major problem in public health and the integration of resources in many countries [1,2]. The healing process is a complex arrangement, but well-ordered phases overlap in which highly specialized cells interact with the extracellular matrix to result in growth and tissue repair [3,4].

The holistic understanding of the processes involved in wound healing has expanded our knowledge of cicatrization, which defines the set of directions for therapeutic decisions in nature and the development of products that provide solutions to the problem [5].

A delayed healing process is considered to be a complex therapeutic problem in modern medicine, especially in light of the knowledge that patients with diabetes and other physiological disorders still require differentiated approaches [1,6]. In this context, the pharmaceutical industry has appealed increasingly to new sources of drugs of natural origin [7].

Lectins are proteins that have the ability to bind to carbohydrates and glycoconjugates without altering their structure [8]. These biomolecules serve as a promising alternative in the treatment of skin wounds due to their potential for promoting healing [9,10,11,12]. To understand the different biological activities related to lectins it is necessary to have knowledge about characterization and structure studies. However, in marked contrast to the lectins from higher land plants, marine algal lectins have been isolated and characterized at a much slower pace. Moreover, to date, biochemical and structural information regarding algal lectins is scarce; hence, the functional and phylogenetic classification of these lectins remains obscure [13]. Moreover, lectins from marine algae appear to have enormous potential for use in biochemistry and biomedical sciences. Despite the small number of published studies, these proteins are known to exert several biological activities, including the induction of neutrophil migration *in vitro* and *in vivo* and anti-nociceptive and anti-inflammatory effects [14,15,16,17].

Thus, the aim of the present work was to characterize the lectin isoforms from the marine red alga *Bryothamnion seaforthii* using mass spectrometry and to investigate the healing potential of topical administration of the lectin on surgically induced skin wounds in a murine model.

## 2. Results and Discussion

### 2.1. Amino Acid Sequence of BSL Isoforms

**Figure 1:**
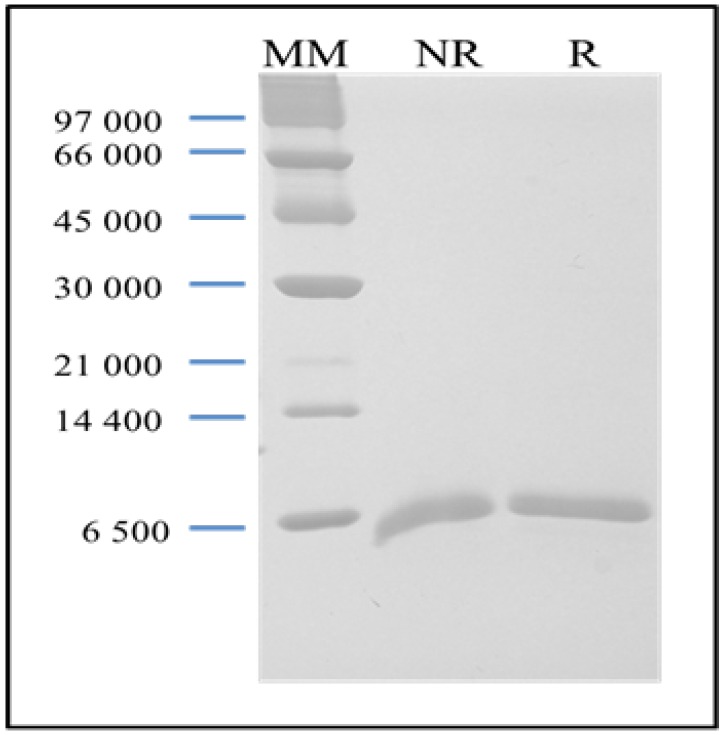
SDS-PAGE of purified BSL. Lane MM: a marker kit including phosphorylase B (97 kDa), BSA (66 kDa), ovalbumin (45 kDa), carbonic anhydrase (30 kDa), trypsinogen (21 kDa), lactalbumin (14.4 kDa) and aprotinin (6.5 kDa). Lane NR: non-reduced BSL. Lane R: reduced BSL.

The pure lectin active fraction was obtained by ion-exchange chromatography as described by Ainouz and co-worker. The hemagglutinating activity of the lectin was inhibited only by the glycoproteins fetuin, avidin and porcine mucin [18]. The purified BSL is observed in SDS-PAGE as a broad band with an apparent molecular mass of 9 kDa in the presence and absence of 2-mercaptoethanol (Figure 1). 

**Figure 2:**
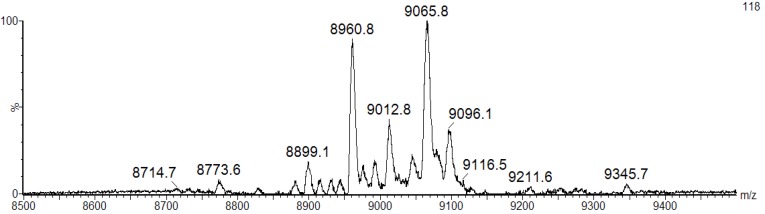
MALDI-TOF-MS analysis of BSL under denaturing conditions. The BSL isoforms exhibit major ion peaks at *m/z* 9065.8 ± 2 and 8960.8 ± 2 and three ion peaks with minor intensity at *m/z* 8899.1 ± 2, 9012.8 ± 2 and 9096.1 ± 2.

We speculate that the broad band pattern could be due to the presence of isomeric forms of BSL with different molecular mass. To confirm the isoforms, the purified BSL was investigated by matrix-assisted laser desorption ionization mass spectrometry (MALDI-MS). The mass spectrometric analysis revealed that the purified BSL contained a mixture of five isoforms, namely: BSL1, BSL2, BSL3, BSL4 and BSL5 at *m/z* 8898.1 ± 2, 8960.8 ± 2, 9012.8 ± 2, 9065.8 ± 2 and 9096.1 ± 2, respectively (Figure 2).

**Figure 3:**
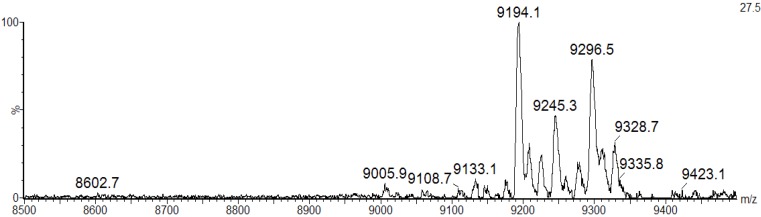
MALDI-TOF-MS analysis of CA-BSL under denaturing and reducing conditions in the presence of iodoacetamide. The BSL samples treated with dithiothreitol (DTT) and iodoacetamide exhibited increased masses at 232 Daltons (4 × 58.00), suggesting the presence of four cysteine residues engaged in the formation of two intrachain disulfide bonds.

The existence of lectin isoforms was also observed in the lectins isolated from *Bryothamnion triquetrum* [19], *Eucheuma serra* [20] and *Hypnea japonica* [21]. The MALDI-TOF masses of intact BSL did not change upon incubation of the lectin with iodoacetamide under non-reducing conditions, indicating that BSL does not possess free sulfhydryl groups (data not shown). In contrast, a MALDI-TOF-MS analysis of denatured, reduced and carbamidomethylated BSL (CA-BSL) exhibited five major ions at *m/z* 9133.1 ± 2, 9194.1 ± 2, 9245.3 ± 2, 9296.5 ± 2 and 9328.7 ± 2, indicating the presence of four cysteine residues involved in two intrachain disulfide bonds (Figure 3). 

These data were confirmed by an analysis of reduced and ethylpyridylated BSL in which the mass increment was 436 Daltons to each native BSL isoform (data not shown). Similar results were obtained for the *Bryothamnion triquetrum* lectin (BTL) and the *Hypnea japonica* lectin (HJA) [19,21]. HML and HCA, lectins isolated from the marine red alga *Hypnea musciformis* and *Hypnea cervicornis*, respectively, have masses of 9 kDa and contain 14 half-cystines each in their amino acid sequences. Furthermore, they are composed of two similar polypeptide chains linked by S–S bonds [13]. The presence of cysteinyl residues in BSL lectins may contribute to their extreme thermostability [22].

**Figure 4:**
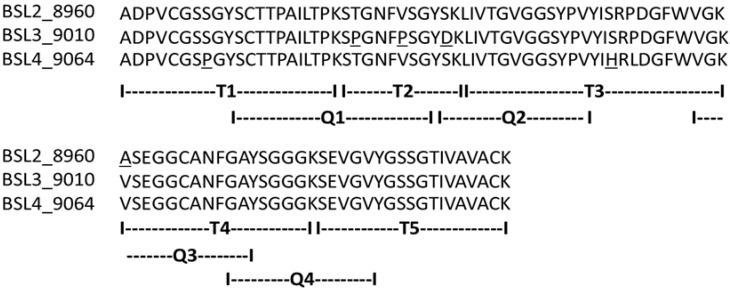
Complete amino acid sequence of BSL 2, BSL 3 and BSL 4. The tryptic and chymotryptic peptides are represented by T and Q, respectively. The underlined residues represent micro heterogeneity of the isoform sequences.

The complete amino acid sequences of BSL2, BSL3 and BSL4 were determined using tandem mass spectrometry (MS/MS) by overlapping the sequences of the peptides generated by digestion of CA-BSL with trypsin and chymotrypsin (Figure 4, Table 1). Sequence heterogeneity was observed at positions 8 (S/P), 23 (T/P), 27 (V/P), 31 (D/S), 47 (S/H), 49 (P/L) and 57 (A/V). 

The isolectins from the marine red alga *Bryothamnion seaforthii* (BSL) are polypeptides composed of 91 amino acid residues including four half-cystines. Methionine and glutamine were not detected in their sequences. The molecules contained a remarkably high content of Ser (13, 12 and 11 in BSL2, BSL3 and BSL4, respectively), Gly (18 in all) and Val (10 in all), indicating that these three amino acids constitute approximately 44% of the total amino acid residues. The isotope-averaged molecular masses calculated for the BSL2, BSL3 and BSL4 isoforms were 8960.28, 9010.309 and 9064.32, respectively, which is in excellent agreement with the experimentally determined masses. Four half-cystines were found in BSL, and no free sulfhydryl groups were identified (Figure 3). The ion at *m/z* 2289.9487 from the pepsin digestion of BSL corresponds to the peptides ^1^ADPVCGSPSGY^11^ and ^53^WVGKASEGGCANF^65^ linked by a disulfide bond between cysteines 5 and 62. The reduction and alkylation of this peptide results in the dissociation of the disulfide bond and the formation of two ions at *m/z* 1430.5880 and 1070.4320 that correspond to the ethylpyridylated peptides WVGKASEGGCANF and ADPVCGSPSGY, respectively. These data suggest that two intrachain disulfide bonds, Cys^5^–Cys^62^ and Cys^12^–Cys^90^, are present in BSL.

**Table 1:** Sequenced peptides and their respective molecular masses.

Peptide	Sequence **	Calculated mass	Experimental mass	Δ Mass (Da)
T1.1 *	ADPVCGSPGYSCTTPAILTPK	2192.99	2192.90	0.09
T1.2 *	ADPVCGSSGYSCTTPAILTPK	2182.88	2182.88	0.00
T2.1	SPGNFPSGYDK	1167.52	1167.43	0.09
T2.2	STGNFVSGYSK	1145.53	1145.50	0.03
T3.1	LIVTGVGGSYPVYISRPDGFWVGK	2566.36	2566.30	0.06
T3.2	LIVTGVGGSYPVYIHRLDGFWVGK	2632.41	2632.19	0.22
T4.1 *	ASEGGCANFGAYSGGGK	1589.68	1589.64	0.04
T4.2 *	VSEGGCANFGAYSGGGK	1617.64	1617.67	−0.03
T5 *	SEVGVYGSSGTIVAVACK	1783.82	1783.87	−0.05
Q1 *	SCTTPAILTP KSPGNFPSGY	2094. 99	2094. 93	0.06
Q2	SKIIVTGVGGSYPVY	1538.83	1538.74	0.09
Q3 *	VGKASEGGCANFGAY	1487.64	1487.60	0.04
Q4	GAYSGGGKSEVGVY	1329.62	1329.55	0.07

* Peptides with carbamidomethylated cysteine; ** All peptides were sequenced using tandem mass spectrometry. The CID spectra were analyzed manually.

**Figure 5:**
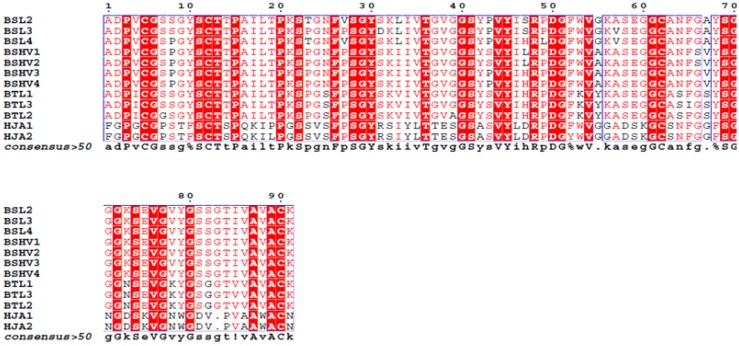
Alignment of the family of lectins similar to BSL. BSL2 to BSL4 are isolectins from *Bryothamnion seaforthii*, BSHV1 to BSHV4 are isolectins from *B. seaforthii* from the Venezuela coast, BTL1 to BTL3 are isolectins from *B. triquetrum*, and HJA1 to HJA2 are isolectins from *Hypnea japonica*.

The alignment of the BSL sequence against the BLAST database reveals 48% identity and 64% similarity between BSL and HJA, a lectin isolated from marine red alga *Hypnea japonica*. Compared with the *B. triquetrum* lectin (BTL), BSL presents only seven exclusive positions, resulting in 83% identity. Relative to BSH, a hemagglutinin isolated from *B. seaforthii* collected on the Venezuela coast [22], BSL diverges in five exclusive positions beyond the heterogeneities among their own isoforms: 43 (P/S), 47 (S/L), 55 (G/A), 66 (G/S) and 67 (A/V) (Figure 5).

In recent years, a large number of lectins have been isolated from marine red alga [23,24,25]. These lectins generally have a low molecular weight, an isoelectric point in the range of pH 4–6, no affinity for simple sugars, no requirement for divalent cations for their biological activities and, in some cases, the presence of repeated domains [24]. Although some of these proteins exhibit potential biological activities [26,27,28,29,30], only a few have actually been studied at the structural level. BTL was the first marine algal lectin to have its primary structure determined [19]. 

In the same year, Hori *et al.* [21] reported the primary structure of HJA, which shares sequence similarity with BTL and constitutes the first marine red alga lectin family. Based on the identity between HML and HCA and the differences in the amino acid sequences compared with BTL/HJA, Nagano *et al.* [13] suggested that HCA/HML constitute a new lectin family. Conversely, the monomeric lectins isolated from *Eucheuma serra*, *E. amakusaensis*, and *E. cottonii* [20] have masses of approximately 28 kDa, exist as monomers, and share *N*-terminal sequence similarity with the complete amino acid sequence of isolectin 2 from *Eucheuma serra* (ESA-2) [31]. 

Additionally, the primary structure of ESA-2 contains repeated domains. These data suggest that lectins from the genus Eucheuma can be grouped in a third family of marine red alga lectins. In the present study, five isoforms of BSL, namely BSL1, BSL2, BSL3, BSL4 and BSL5, were identified, and the amino acid sequence of three (BSL2, BSL3, and BSL4) were determined from an MS/MS analysis of tryptic and chymotryptic peptides. BSL exhibited four cysteines in its primary structure that are involved in two intrachain disulfide bonds: Cys^5^–Cys^12^ and Cys^62^–Cys^90^. However, in BTL, one of these cysteines can interact with a cysteine present in another polypeptide chain to eventually form a dimer [19]. In HJA, the disulfide bonds are exclusively involved in intrachain bonds with the pattern Cys^5^–Cys^62^ and Cys^12^–Cys^89^[21]. The data obtained with BSL suggest that the disulfide bonds are also exclusively intrachain with the same pattern observed in HJA. Although the pattern of disulfide bonds between HJA and BSL is different from that presented by BTL, given the similarity between these lectins and the presence of isoforms among other similar biochemical characteristics, the data strongly suggest that BSL is a novel member of the first marine red alga lectin family that includes the isoforms of HJA, BTL and BSH.

### 2.2. Evaluation of Healing Potential

It was observed that the treatment of wounds with the lectin isolated from the marine red alga *Bryothamnion seaforthii* (BSL) induced a greater inflammatory process, which is observed during clinical evaluation and by well-evidenced phlogistic signs, such as edema and hyperemia in the first days following treatment, when compared with controls. Such signs are indicative of a pro-inflammatory effect by the lectin tested (data not shown).

**Figure 6:**
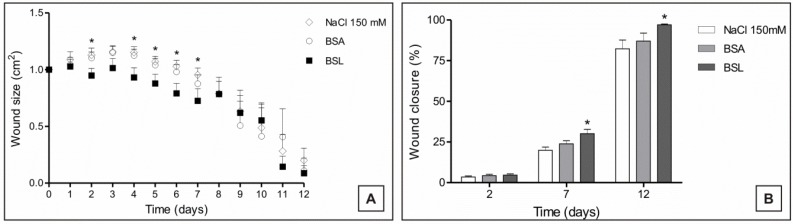
(**A**) Evolution of the areas; and (**B**) proportion of experimental closure of skin lesions in mice treated topically with 150 mM NaCl, bovine serum albumin (BSA) and *Bryothamnion seaforthii* lectin (BSL); * Significant in relation to the 150 mM NaCl group, *p* < 0.05.

The measurement of the areas of the experimental lesions was performed daily to observe the evolution of healing in the experimental groups. During the inflammatory and proliferative phase, it could be observed that treatment with BSL induced a decrease in the areas of the lesions of the experimental animals in this group (Figure 6).

The percentage of wound closure was evaluated from the exposed areas of each lesion according to the time. BSL treatment induced rapid and more effective healing compared with the controls (BSA and 150 mM NaCl) in which a higher proportion of closure in the animals treated with the lectin was observed on POD (postoperative day) 2, 7 and 12 (Figure 6). Thus, the greater effectiveness of treatment with BSL can be observed in the proliferative and remodeling phases of the healing process when compared to the controls.

The BSL treatment induced an increased closing in the areas of the lesions in the group treated with BSL from POD 1 to 12, and this was possibly influenced by the contraction process of the wounds during healing.

### 2.3. Histopathological Assessment

For the histopathological evaluation of the injured tissue, samples were collected from each group on POD 2, 7 and 12 (*n* = 4 for all groups). 

**Figure 7:**
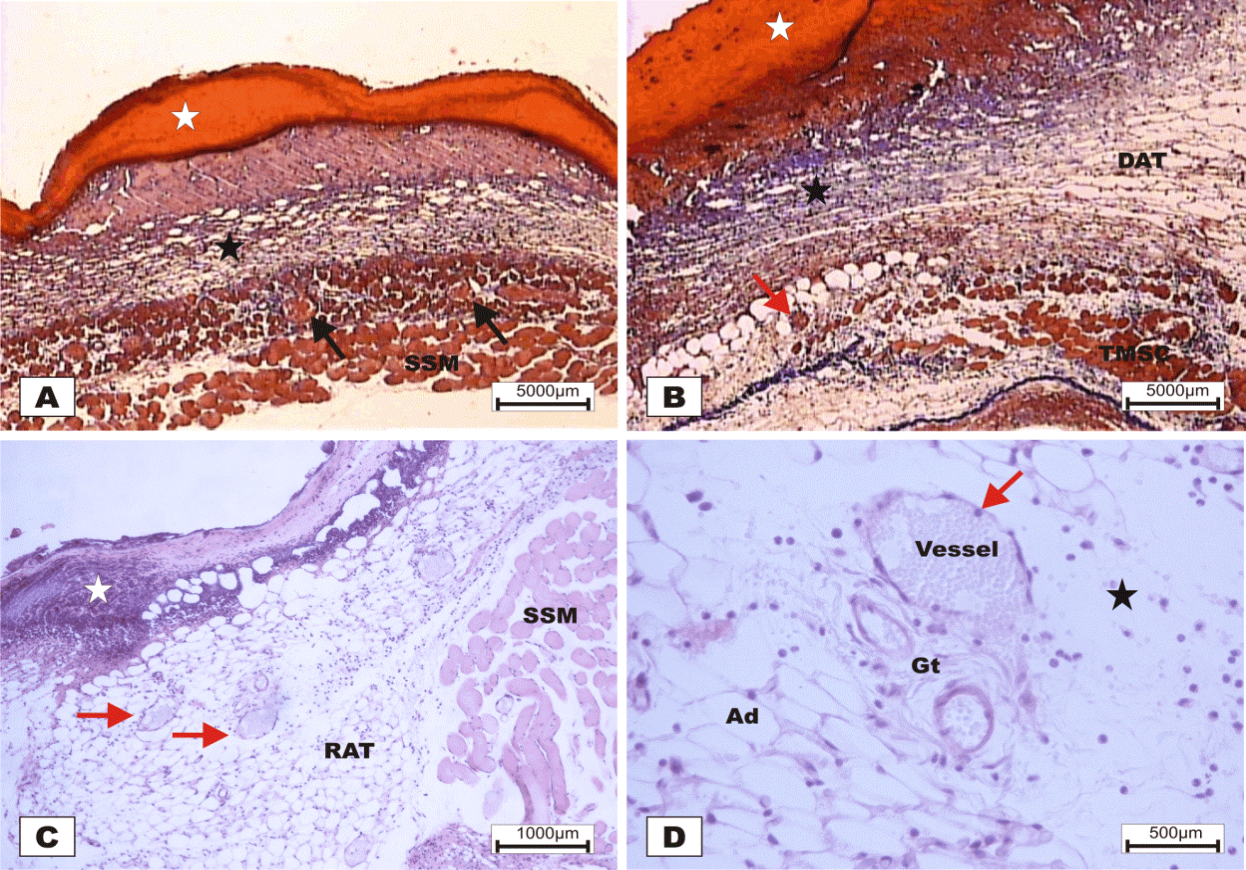
Photomicrographs of surgically induced skin wounds in mice treated with a topical administration of BSA and BSL in POD 2 (Stain: hematoxylin-eosin and Masson’s trichrome). (**A**) (BSA). Note the presence of a scab (white star) covering the wound and intense inflammatory exudate. Below the exudate, a collagenolysis area and edema of the reticular dermis are observed (black star). Immediately below the dermis is the subcutaneous muscle layer with congested vessels (arrows). A 4× objective was used; (**B**) (BSA). Note the presence of a thicker crust (white star) covering the wound bed and a less-intense inflammatory exudate. Below the exudate, a collagenolysis area, edema of the reticular dermis and intense inflammatory infiltrate are observed (black star) with degeneration fatty tissue (DAT). Note the muscle fibers spaced by muscle tissue and fat cells with congested vessels near the collagenolysis area (red arrow). A 4× objective was used; (**C**) (BSL). A thick scab covers the wound bed (white star). Note the congested vessels of the granulation tissue (arrow) in the reactional adipose tissue (RAT). A 10× objective was used; (**D**) (BSL). Detail from the previous photo showing vessels of the granulation tissue in areas of intense inflammatory exudate (star). Observe the possible diapedesis of the neutrophil PMNs into the inflamed area (red arrow). A 40× objective was used.

The study revealed that the wounds treated with BSA and BSL exhibited scabs sealing the epithelium opening on POD 2 (Figure 7). During the histopathological diagnosis, wounds treated with BSL showed intense inflammatory exudates in the reactional adipose tissue, with a mild inflammatory infiltrate in the reticular dermis in addition to the presence of granulation tissue in the reactional adipose tissue (Figure 7C,D). In wounds treated with BSA, there were intense inflammatory exudates, collagenolysis, and edema of the reticular dermis with the degenerative adipose tissue showing the beginning of a poor healing (Figure 7A,B).

**Figure 8:**
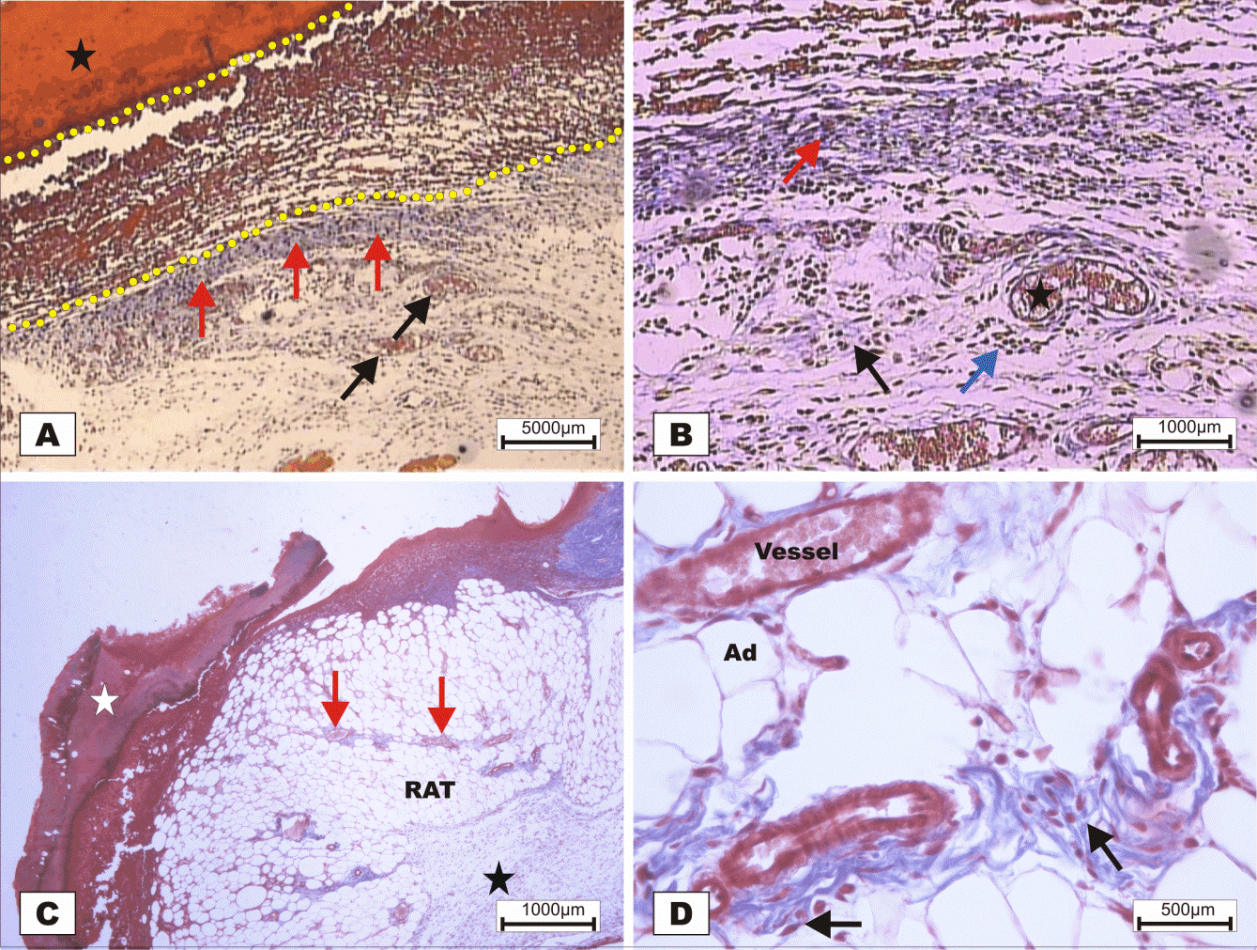
Photomicrographs of surgically induced skin wounds in mice treated with a topical administration of BSA and BSL in POD 7 (Stain: Masson’s trichrome). (**A**) (BSA). Note the presence of a scab (star) covering the wound bed and intense inflammatory exudate (delimited by dashed yellow lines). Below the exudate, observe the area of granulation tissue with congested vessels (arrow) and intense fibroblastic proliferation. Immediately beneath the inflammatory exudate, note the bluish band resulting from the synthesis of collagen (red arrows). A 4× objective was used; (**B**) (BSA). Details of the granulation tissue. Note the fibroblastic proliferation (arrow) and mild inflammatory infiltrate around the vessels (blue arrow). Note the collagen above the range of the granulation tissue (red arrow). Vessels of the granulation tissue are observed (star). A 10× objective was used; (**C**) (BSL). Overview of the injured area. Note the presence of a crust sealing the epithelial opening (white star). Vessels of the granulation tissue in the reactional adipose tissue (RAT) surrounded by thickening collagen fibers (arrows) resulted from intense fibroblastic synthesis. An area with a mild inflammatory exudate is observed (black star). A 10× objective was used; (**D**) (BSL). Detail from the previous photo showing the area of granulation tissue vessels surrounded by active collagen. Note the active fibroblasts in the area (arrows). A 40× objective was used.

On POD 7, densification of collagen fibers in the reticular dermis, which is primarily surrounded by the vessels of the granulation tissue in adipose tissue with the presence of discrete reactional inflammatory exudates, was observed (Figure 8C,D). In the group treated with BSA (Figure 8A,B), intense inflammatory exudates and early fibroblastic proliferation with moderate collagen synthesis were still observed, demonstrating a less evolved healing process when compared with the wounds treated with BSL.

**Figure 9:**
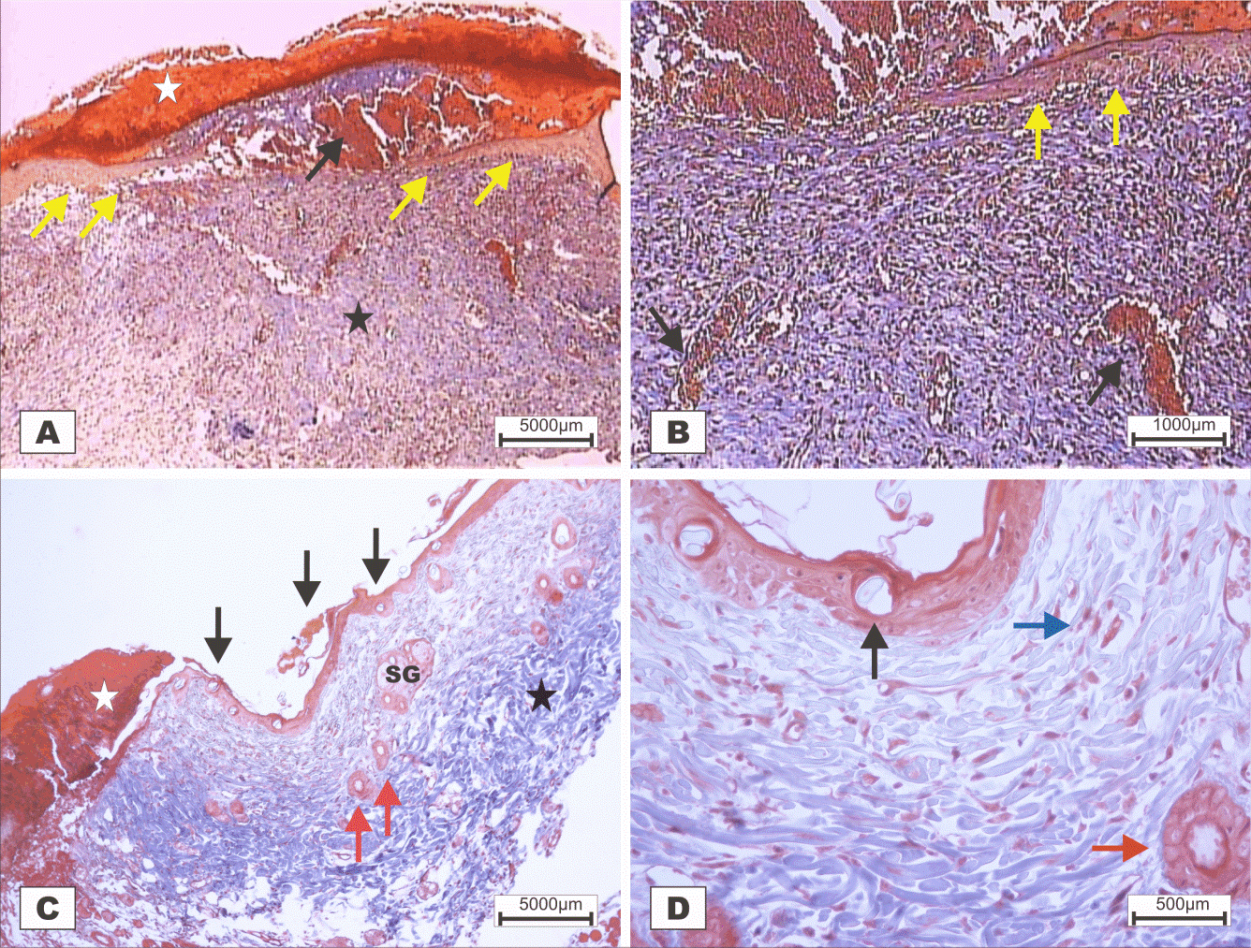
Photomicrographs of surgically induced skin wounds in mice treated with a topical administration of BSA and BSL in POD 12 (Stain: Masson’s trichrome). (**A**) (BSA). Presence of a scab (white star) and an inflammatory exudate beneath the scab (arrow). Note the progression of the luminal epithelium (yellow arrows) below the crust and inflammatory exudate. Dermis with less collagen deposition and the presence of active granulation tissue (black star) in a regression process is observed. A 4× objective was used; (**B**) (BSA). Details of the previous photo. Observe the granulation tissue characterized by the presence of vessels (black arrows) and young collagen in the extracellular matrix with inflammatory infiltrate. Note the projection of the epithelium beneath the crust (yellow arrows). A 10× objective was used; (**C**) (BSL). Note also the newly formed epithelium coated with little production of keratin (black arrows). The presence of a fragment of crust on the edge of the injured area (white star), the formation of skin appendages as sebaceous glands (SG), and the remaining vessels of the granulation tissue can be observed (red arrows). Note the active collagen in the reticular dermis (black star). A 4× objective was used; (**D**) (BSL). Details of the previous photo showing the presence of cutaneous annexes in the area of the newly formed epithelium (black arrow), fibroblasts in the papillary dermis area (blue arrow) and characteristic neoangiogenesis vessels in the granulation tissue (red arrow). A 40× objective was used.

At POD 12, the BSL-treated animals experienced restructuring of the epithelium covering with little production of keratin, formation of cutaneous immature annexes, and active collagen in the region of the reticular dermis with presence of fibroblastic activity (Figure 9C,D). In the lesions treated with BSA over the same period, an epithelial scab sealing the epithelium opening, a mild inflammatory infiltrate beneath the crust and progression of the epithelial lining were observed (Figure 9A,B).

**Figure 10:**
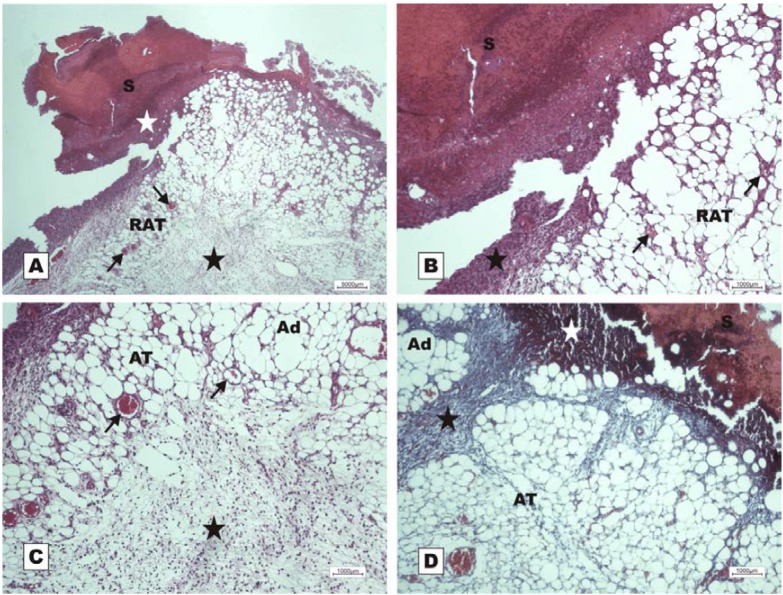
Photomicrographs of surgically induced skin wounds in mice treated with a topical administration of 150 mM NaCl in POD 7 (Stain: hematoxylin-eosin and Masson’s trichrome). (**A**) (NaCl). Open wound with a thick scab and dystrophic calcification (white star) sealing the epithelial opening (S). Observe the beginning of granulation tissue formation originating from the reactional adipose tissue (RAT), and note the presence of congested vessels (arrows) and the area of inflammation (star). A 4× objective was used; (**B**) (NaCl). Details showing the congested vessels in fat tissue (arrows) and the inflammatory infiltrate beneath the crust (star). A 10× objective was used; (**C**) (NaCl). Details of the area of acute inflammation in the degenerative adipose tissue (star) and the granulation tissue vessels above the inflamed area (arrows). A 10× objective was used; (**D**) (NaCl). Observe the inflammatory exudate beneath the crust (white star). There is poor collagen deposition and poor formation of granulation tissue in the dermis (black star) between the areas of adipose tissue. A 10× objective was used.

In the control group treated with 150 mM NaCl, there was a delay in wound healing in which, at POD 7, acute inflammation and granulation tissue at the beginning of proliferation were observed (Figure 10).

Wound healing is characterized as a complex process that results in contraction and wound closure, as well as restoration of the epithelium as a functional coating [32]. Thus, the repair of skin wounds is accompanied by an ordered sequence of biological events starting with the closure of the wound and advancing to the remodeling of damaged tissue [33].

The use of natural products to improve and accelerate the healing process has been widely envisioned because delayed skin healing, such as a problem in tissue repair in individuals suffering from diabetes or leprosy, is a major economic implication in medicine. This economic factor is a result of large drug spending over a long period that patients who suffer from chronic wounds or ulcers spend in hospitals [1,6,34]. Thus, agents that decrease the time of the tissue repair process are desired to contribute to a faster and more effective healing process [35].

Lectins are a structurally heterogeneous group of proteins of non-immune origin that have the characteristic to bind carbohydrates reversibly with high selectivity [36]. Lectins are very versatile proteins and have many direct applications in biological and biomedical research [37].

The field of application of lectins in the treatment of wounds is unexplored. However, important studies have shown that these biomolecules have healing potential in the treatment of acute wounds [11,12].

The biological application of lectins from marine algae deserves special interest because these lectins are lower molecular weight proteins than plant lectins. Thus, it is expected that these marine algae lectins are much less antigenic than others [38]. Additionally, lectins from marine algae seem to express their biological activities in a stronger manner than lectins from other sources. Furthermore, they possess great molecular stability due to several disulfide bridges and show high specificity for complex carbohydrates and glycoconjugates, especially for mucins [13,21,39].

The lectin isolated from the marine red alga *Bryothamnion seaforthii* has been shown to have several biological activities, as described in the literature [14,27,40,41], and may be considered to be an important tool for biomedical applications.

Our findings demonstrate the potential pro-healing activity of the BSL, which was responsible for acceleration in the healing of skin wounds treated with this lectin. Thus, the histopathological findings showed that the lectin used in this study exerts its activity on the migration of polymorphonuclear cells to the injured site, confirming the data already published [11] that suggest that the lectin of *Artocarpus integrifolia* (KM+) induced a more effective healing of the corneal epithelium via a mechanism that also involves the migration of neutrophils.

It is possible that BSL is also able to act on the activation and proliferation of fibroblasts because most of the lectin-treated animals exhibited strongly active collagen in the dermis. However, future experiments are needed to better characterize the action of the BSL on fibroblasts.

## 3. Experimental Section

### 3.1. Algal Collection and Lectin Purification

Specimens of the marine red alga *B. seaforthii* were collected at the northeast coast of Brazil (Pacheco beach, Ceará). The material was cleansed from epiphytes and stored at −20 °C until it was used. The purification of BSL was performed according to the method of Ainouz *et al.* [18]. The frozen algae were thawed, rinsed with distilled water, ground to a ﬁne powder under liquid nitrogen, extracted with three volumes of 20 mM sodium phosphate, pH 7.0, containing 150 mM NaCl for 4 h while stirring, ﬁltered through nylon tissue and centrifuged at 7000× *g* for 30 min at 4 °C. The supernatant was acidiﬁed and maintained at 4 °C for 4 h. The precipitates were removed by centrifugation at 15000× *g* for 20 min at 4 °C, and the supernatant was adjusted to pH 7.0 and subjected to fractionated precipitation with 60% ammonium sulfate saturation. The precipitated proteins were pelleted by centrifugation, resuspended in a small volume of 20 mM phosphate buffer (PB) pH 7.0, and applied to a DEAE-cellulose column. The column was equilibrated and washed with PB, and this was followed by elution with 1 M NaCl in the same buffer. Fractions of 2 mL were manually collected at a flow rate of 1 mL·min^−1^ and analyzed using a spectrophotometer to measure the absorbance at 280 nm and tested for hemagglutinating activity. The unadsorbed fractions were rechromatographed on the same column, pooled, dialyzed against water and lyophilized until further use.

### 3.2. Protein Analytical Methods

The protein purity and homogeneity were achieved using SDS-PAGE [42]. The molecular mass was determined using MALDI-TOF mass spectrometry with a SYNAPT HDMS instrument operating at a 20 kV accelerating voltage in the reflector V-mode. Sinapinic acid (3,5-dimethoxy-4-hydroxycinnamic acid) saturated in 50% acetonitrile and 0.1% trifluoracetic acid (TFA) was used as sample matrix.

### 3.3. Determination of Sulfhydryl Groups and Disulfide Bonds

For the quantization of free cysteine residues in BSL, the lectin was solubilized in 10 mM ammonium bicarbonate, incubated with either 5 mM iodoacetamide or 5 mM 4-vinylpyridine for 1 h at room temperature and subjected to MALDI-TOF-MS. The number of free cysteine residues (*N*_SH_) was determined using the following equation:


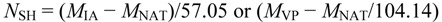


*M*_IA_ is the mass of the non-reduced protein in the presence of iodoacetamide, *M*_VP_ is the mass of the non-reduced protein in the presence of 4-vinylpyridine, *M*_NAT_ is the mass of the native protein, 57.05 is the mass increment due to the carbamidomethylation of one thiol group and 104.14 is the mass increment due to the ethylpyridylation of one thiol group.

For the quantization of the disulfide bonds, BSL was incubated with 1 mM DTT followed by the addition of five-fold molar excess of 4-vinylpyridine or iodoacetamide as alkylating agents. The samples were incubated for 1 h at room temperature before being subjected to MALDI-TOF-MS analysis. The number of total cysteine residues (*N*_Cys_) was derived using the following equation:


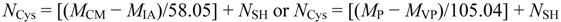


M_CM_ is the mass of the reduced and carbamidomethylated protein, and 58.05 is the mass increment due to the carbamidomethylation of a cysteine residue. MP is the mass of the reduced and ethylpyridylated protein, and 105.04 is the mass increment due to the ethylpyridylation of a thiol group, which was involved in the formation of a disulfide bond prior to reduction.

Lastly, the number of disulfide bonds (*N*_S–S_) was calculated as follows:


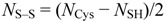


All mass values are in Daltons.

To determine the positions of disulfide bonds, native BSL was digested with pepsin in 10 mM HCl containing 1.5 M guanidine hydrochloride at 37 °C for 18 h. The peptides produced were separated using HPLC with a Sephasil C18 column at flow rate of 1 mL·min^−1^. The 1 mL fractions were collected and analyzed using MALDI-TOF-MS. The peptides containing disulfide bonds were reduced and alkylated to confirm the position of the cysteines involved in the S–S bonds.

### 3.4. Enzymatic Digestion and Mass Spectrometry Analysis

The Coomassie stained protein bands of BSL were excised from the SDS-PAGE gel and subjected to tryptic and α-chymotryptic digestion overnight at 37 °C at an enzyme: substrate ratio of 1:50 (w/w). Briefly, the excised gel pieces were washed with 100 mM ammonium bicarbonate (NH_4_HCO_3_) and 50% acetonitrile until the gel pieces appeared colorless. The gel pieces were then dehydrated in 100% acetonitrile. Reduction of protein was performed in 10 mM DTT containing 50 mM ammonium bicarbonate at 56 °C for 45 min. Alkylation of protein was performed with 55 mM iodoacetamide in 50 mM ammonium bicarbonate at room temperature for 1 h. The gel pieces were washed three times with 100 mM ammonium bicarbonate in 50% acetonitrile, dehydrated in 100% acetonitrile and dried in a speed vac. A trypsin plus α-chymotrypsin solution was prepared in 50 mM NH_4_HCO_3_, and 20 μL of this solution was added to each tube and stored at 4 °C for absorption analysis. When needed, more NH_4_HCO_3_ was added until that the gel was completely immersed.

The digestion was stopped by the addition of 2% formic acid. The gel pieces were washed four times with 5% formic acid in 50% acetonitrile. The supernatants were then collected and transferred to fresh tubes, pooled, dried in a speed vac and resolubilized in 20 μL of 0.1% formic acid. Finally, 2 μL of the peptide solution was chromatographed on a C-18 (0.075 × 100 mm) nano column coupled to a nanoACQUITY system. The column was equilibrated with 0.1% formic acid and eluted with a 10% to 85% acetonitrile gradient containing 0.1% formic acid. The eluates were directly infused in the electrospray source of a mass spectrometer instrument (Synapt HDMS). The mass spectrometer operated in positive mode with a source temperature of 363 K and a capillary voltage of 3.0 kV. The LC-MS/MS was performed according to DDA (data dependent acquisition). The selected precursor ions were fragmented by collision-induced dissociation (CID). The CID spectra were interpreted manually. 

### 3.5. Bioinformatics Analysis

The amino acid sequence similarity searches were performed against a non-redundant protein databank using BLAST. The alignments were performed using ESPript 2.2 [43].

### 3.6. Induction and Treatment of Skin Wounds

The *in vivo* study employed 84 ten-week-old male Swiss albino mice (*Mus musculus*) weighing 35.0 ± 5.0 g that were supplied by the laboratory animal facility of the Federal University of Ceará (BIOCEN/UFC). During the experimental procedures, the animals were kept in individual cages in a controlled environment (circadian cycle, 25 ± 2 °C, 55 ± 10% humidity, food and water *ad libitum*) at the School of Medicine, Sobral (UFC).

Prior to the surgical procedure, the animals were randomly distributed into three groups (*n* = 12, each group with 4 animals) according to the topical treatment administered: G-I (200 µg/mL BSL); G-II (200 µg/mL BSA); and G-III (150 mM NaCl).

The mice were then anesthetized using a subcutaneous administration of 2% xylazine with 10% ketamine hydrochloride (10 mg/kg and 115 mg/kg, respectively) [44], and this was followed by trichotomy and antisepsis of the dorsal thoracic region with povidone-iodine and a sterile saline solution (150 mM NaCl). After marking the skin with a sterile mold (1.00 cm^2^), circular aseptic skin wounds were created by incision with a scalpel (#15) followed by resection of the subcutaneous tissue with fine dissection tweezers. 

Immediately after the surgical procedure, the wounds were topically treated during 12 days with a single dose per day containing 100 µL of the respective substance (BSL, BSA or NaCl) as previously mentioned.

The experimental protocol was approved by the Ethics Committee of Ceara State University (UECE) under entry #11042434-4, and all animals were treated according to the recommendations of the Brazilian College of Animal Experimentation (COBEA) and the Guide for the Care and Use of Laboratory Animals of the US Department of Health and Human Services (NIH publication No. 85–23, revised 1985).

### 3.7. Evaluation of Healing Potential

The wounds were measured daily for 12 days to evaluate the healing potential of each treatment. The wound area was expressed as the mean ± standard deviation, as previously reported [45].

The animals were anesthetized prior to the collection of histopathological material and subsequently euthanized [44]. Samples (4 per group) of injured tissue were collected on the 2nd, 7th and 12th postoperative day (POD), fixed in 10% formaldehyde (v/v) buffered in 0.01 M PBS (pH 7.2), prepared in 5 mm cuts for routine histological analysis and stained with hematoxylin-eosin (HE) and Masson’s trichrome. The histopathological assessment included the following parameters: the presence of scabs, re-epithelialization, collagen deposition, neovascularization and exudate. 

The analysis was performed under a Leica light microscope (model DM 500) at 4, 10 and 40× magnification.

### 3.8. Statistical Assessment

Differences between the wound area and closure percentage were tested with the Mann-Whitney test. The data were processed using the statistics software GraphPad Prism v.3.00 for Windows^®^. The values are given as the mean ± standard deviation. The level of statistical significance was set at *p* < 0.05.

## 4. Conclusions

Our findings demonstrate the presence of isoforms with biochemical characteristics similar to other lectins from marine algae. These results placed BSL in the group of marine algae that possess isoforms. Treatment with BSL (native lectin) in induced skin wounds in mice can stimulate the healing process, possibly by inducing immune system cells in the inflammatory phase, resulting in the modulation of growth factors and cytokines, as well as stimulating collagen synthesis by fibroblasts and their differentiation into myofibroblasts for traction and wound contraction, which promotes faster closure of the lesion.

However, further studies are required to confirm the role of the lectin used on cells and the growth factors involved in the healing process, as well as possible mechanism of action of the lectins. 
